# The complete chloroplast genome and phylogenetic analysis of *Astragalus floridulus* Podlech 1993 (Fabaceae)

**DOI:** 10.1080/23802359.2026.2753255

**Published:** 2026-11-02

**Authors:** Qiu Quan, Jiehua Rao, Kailing Wang, Chunxiang Jiao, Yongzeng Zhang

**Affiliations:** ^a^ Yunnan Key Laboratory of Screening and Research on Anti-pathogenic Plant Resources from Western Yunnan, Dali, China; ^b^ College of Pharmacy, Dali University, Dali, China

**Keywords:** *Astragalus floridulus*, IRLC, complete chloroplast genome, phylogenetic analysis

## Abstract

*Astragalus floridulus* Podlech is a perennial herb endemic to alpine habitats. We sequenced and characterized its complete chloroplast genome and clarified its phylogenetic placement within the genus. The complete chloroplast genome of *A. floridulus* is 122,371 bp long, with a GC content of 34.19%, and lacks one copy of the inverted repeat (IR) region. It contains 110 genes, comprising 76 protein-coding genes, 30 tRNA genes, and 4 rRNA genes. Phylogenetic analysis indicated that *A. floridulus* is closely related to *A. yunnanensis.* These findings provide baseline genomic data for species identification and taxonomic research on *A. floridulus*.

## 1. Introduction

In the subfamily Papilionoideae (Fabaceae), the lineage characterized by the loss of one copy of the inverted repeat (IR) in the chloroplast genome is known as the Inverted Repeat-Lacking Clade (IRLC) (Lee et al. 2021; Sabir et al. 2014). This clade encompasses approximately 48 genera and over 4,000 species, with key representative genera including *Astragalus*, *Caragana*, *Chesneya*, *Hedysarum*, and *Vicia* (Duan et al. 2021; Wojciechowski et al. 2004).

*Astragalus* is one of the largest genera of vascular plants, comprising approximately 3,000 species globally, of which 401 have been recorded in China (Xu and Podlech 2010). *Astragalus* species have been used in traditional Chinese medicine for over 2,000 years (Wang et al. 2023) and contain flavonoids, saponins, polysaccharides, and amino acids (Klichkhanov and Suleimanova 2024; Li et al. 2025). As of August 2026, only 72 *Astragalus* species had complete chloroplast genomes deposited in NCBI, indicating limited taxonomic sampling.

*Astragalus floridulus* Podlech 1993 is mainly distributed in Gansu, Qinghai, Sichuan, and the Tibet Autonomous Region of China, growing in alpine grasslands or thickets at 2,600 to 4,300 m (Xu and Podlech 2010). Its phytochemistry, pharmacology, and taxonomy remain poorly studied (Liu et al. 2017; Pan et al. 1996). To address these gaps, we sequenced and analyzed the chloroplast genome of *A. floridulus* and clarified its phylogeny, aiming to provide fundamental data for future research and resource utilization of this species.

## 2. Materials and methods

### 2.1. Sampling

Samples of *Astragalus floridulus* were collected from Dong’eluo Second Village, Xinduqiao Town, Kangding City, Sichuan Province, China (30°02’43.861ʺN, 101°29’35.926ʺE) by Dr. Yongzeng Zhang on 31 July 2025 (Figure 1). Leaves were dried with silica gel for subsequent analysis. The voucher specimen has been deposited in the Yunnan Key Laboratory of Screening and Research on Anti-pathogenic Plant Resources from Western Yunnan (voucher code: 20250731-1, contact: Yongzeng Zhang, cell_zyz@163.com).

**Figure 1.**
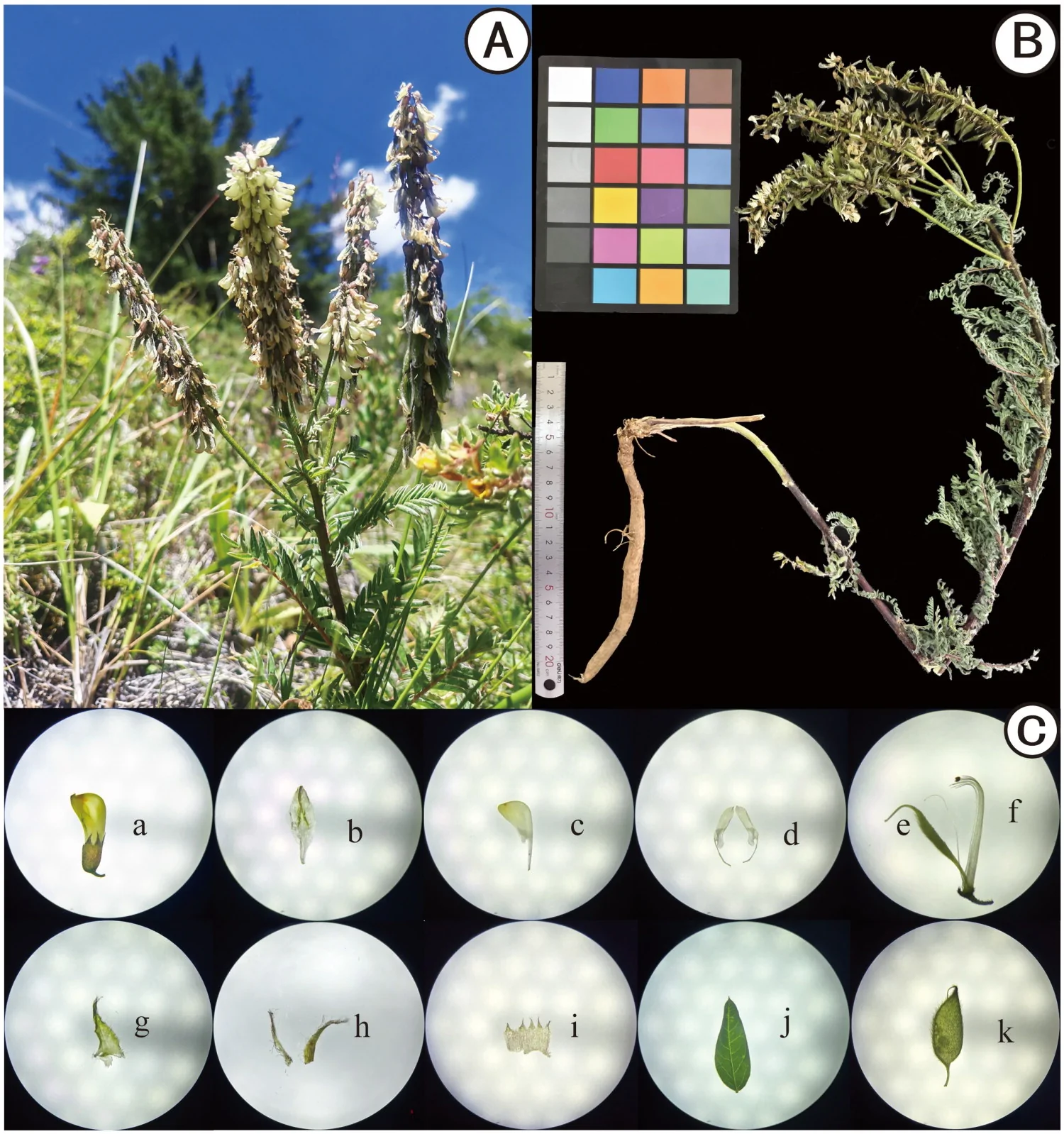
*Astragalus floridulus* Podlech. Photographs were taken by Yongzeng Zhang in Dong’eluo Second Village, Sichuan Province, China. (A) Habit, showing the overall growth form in its natural habitat; (B) A single plant individual; (C) Morphological details of floral and vegetative organs: (a) corolla yellow; (b) standard narrowly oblong-elliptic; (c) keel semi-ovate, with short auricles; (d) wings with short auricles; (e) ovary linear, densely covered with black or intermixed white pubescence; (f) androecium diadelphous (9 + 1); (g) stipule lanceolate or narrowly triangular; (h) bracts lanceolate; (i) calyx with outer surface and inner surface of teeth covered with black appressed hairs, teeth subulate; (j) leaflet linear-lanceolate or oblong, green above, nearly glabrous, grayish-white pubescent below; (k) legume ellipsoid, surface covered with brown or black hairs.

### 2.2. DNA extraction and Sequencing

Chloroplast genomic DNA was extracted using a modified CTAB method (Doyle and Doyle 1987), and quality-checked by agarose gel electrophoresis and spectrophotometry. DNA was sheared into 350 bp fragments for library construction and sequenced on the DNBSEQ-T7 platform. Low-quality sequences were removed using fastp v0.23.2 (Chen 2023), and sequencing depth was assessed with SAMtools v1.18 (Li et al. 2009). Sequencing was performed by Benagen Technology Co. (Wuhan, China).

### 2.3. Genome assembly and annotation

The complete chloroplast genome of *Astragalus floridulus* was assembled using GetOrganelle v1.7.7.1 (Jin et al. 2020) with a maximum of 15 extension rounds, k-mer sizes of 21, 45, 65, 85, and 105, and embplant_pt as the target organelle database. The assembled genome was annotated using CPGAVAS2 (Shi et al. 2019) and visualized with OGDRAW v1.3.1 (Greiner et al. 2019). tRNA genes were identified using tRNAscan-SE v2.0.11 (Chan et al. 2021) in organelle mode, which applies organelle-specific covariance models for tRNA search. rRNA genes were detected using BLASTN v2.14.0 (Chen et al. 2015). The annotated genome was submitted to GenBank under accession number PZ364413.1.

### 2.4. Phylogenetic analysis

We combined our newly sequenced and assembled complete chloroplast genome of *Astragalus floridulus* from this study with publicly available Fabaceae chloroplast genomes from the NCBI database. The final dataset contained 37 accessions representing 36 Fabaceae species: 28 accessions covering 27 *Astragalus* species, including our sample and previously published *A. floridulus* isolate xz41 (accession number PZ369833.1); six *Oxytropis* species; and three outgroup species, namely *Glycyrrhiza glabra*, *Caragana sinica*, and *C. rosea*. Sequences were aligned using MAFFT v7.313 (Katoh and Standley 2013) and trimmed using trimAl v1.4 (Capella-Gutiérrez et al. 2009). Phylogenetic trees were constructed using Bayesian inference (BI) and maximum likelihood (ML). The best-fit substitution models selected by ModelFinder (Kalyaanamoorthy et al. 2017) in PhyloSuite v1.2.3 (Zhang et al. 2020) were GTR+F + G4 (BI) and GTR+F + R4 (ML). BI analysis used MrBayes v3.2.7 (Ronquist et al. 2012) with two parallel runs and 1,000,000 generations, discarding the first 25% as burn-in. ML analysis used IQ-TREE v2.2.0 (Nguyen et al. 2015) with 1,000 ultrafast bootstrap replicates. Phylogenetic trees were visualized in FigTree v1.4.4 (Rambaut 2018) and edited using Adobe Illustrator 2020.

## 3. Results

The complete chloroplast genome of *Astragalus floridulus* was circular, 122,371 bp in length, with a GC content of 34.19% (Figure 2). It lacked one IR region, consistent with the structural characteristics of the IRLC. The genome contained 110 genes, including 76 protein-coding genes, 30 tRNA genes, and 4 rRNA genes (Table S1). Sequencing depth ranged from 185× to 2,088×, averaging approximately 1,689× (Figure S1), with complete and uniform coverage, ensuring reliable assembly and annotation. We identified 10 cis-splicing genes (*ycf3*, *rpoC1*, *atpF*, *clpP*, *petB*, *petD*, *rpl16*, *rpl2*, *ndhB*, *ndhA*; Figure S2) and one trans-splicing gene (*rps12*; Figure S3).

**Figure 2.**
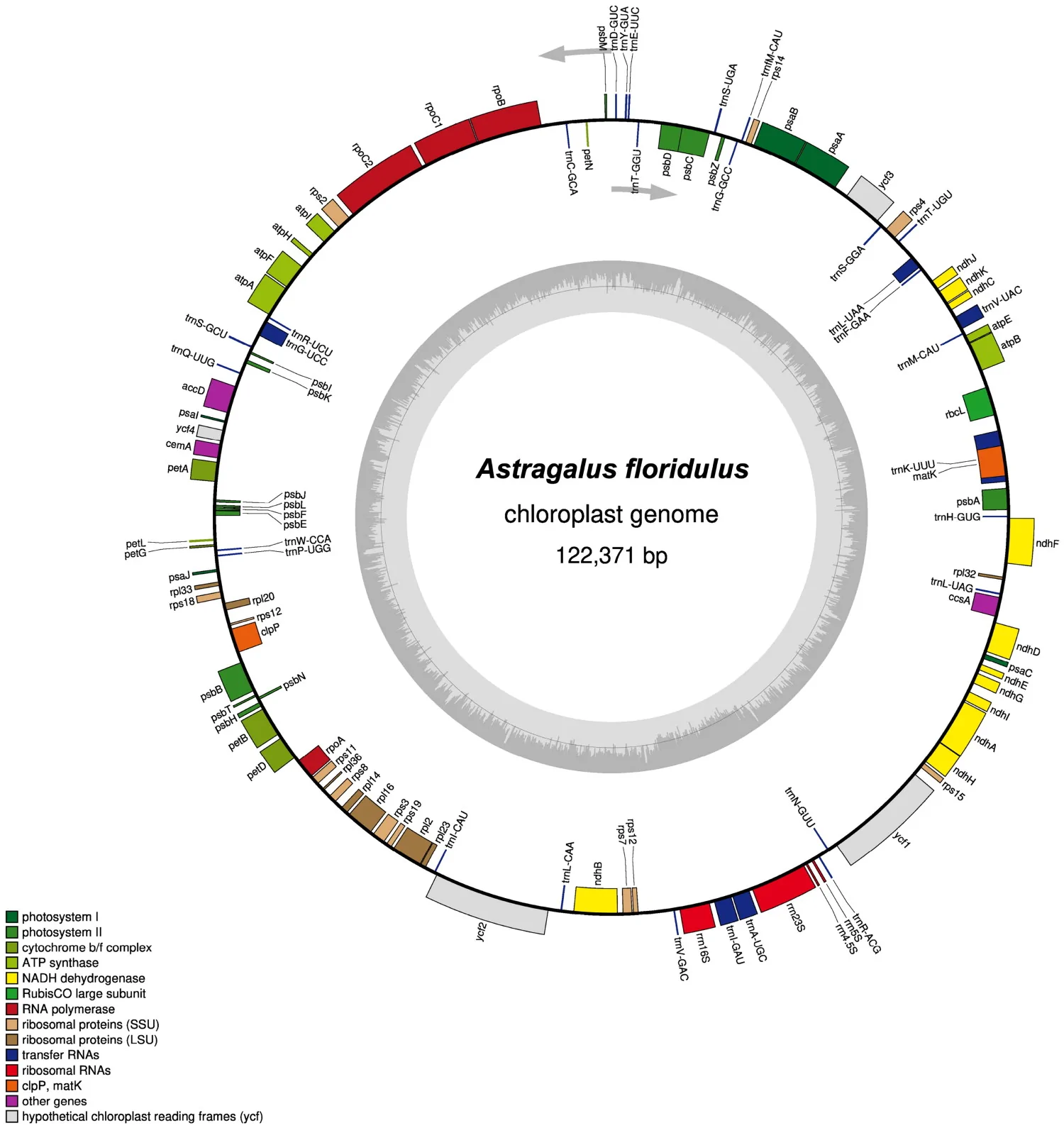
Circular map of the *Astragalus floridulus* chloroplast genome. Genes drawn inside the circle are transcribed clockwise, whereas those outside are transcribed counterclockwise. Different functional gene categories are represented by different colors. The dashed area in the inner circle indicates the GC content.

ML and BI phylogenetic trees based on complete chloroplast genomes showed congruent topologies with high statistical support. The phylogenetic analysis revealed that *Astragalus* and *Oxytropis* formed distinct monophyletic groups (BS = 100%, PP = 1.0, Figure 3), while the outgroup taxa fell outside the *Astragalus-Oxytropis* clade. Within the *Astragalus* clade, our *A. floridulus* sample clustered closely with *A. floridulus* isolate xz41, indicating high sequence identity. The two *A. floridulus* accessions and *A. yunnanensis* together formed a highly supported subclade (BS = 100%, PP = 1.0, Figure 3), suggesting a close phylogenetic relationship.

**Figure 3.**
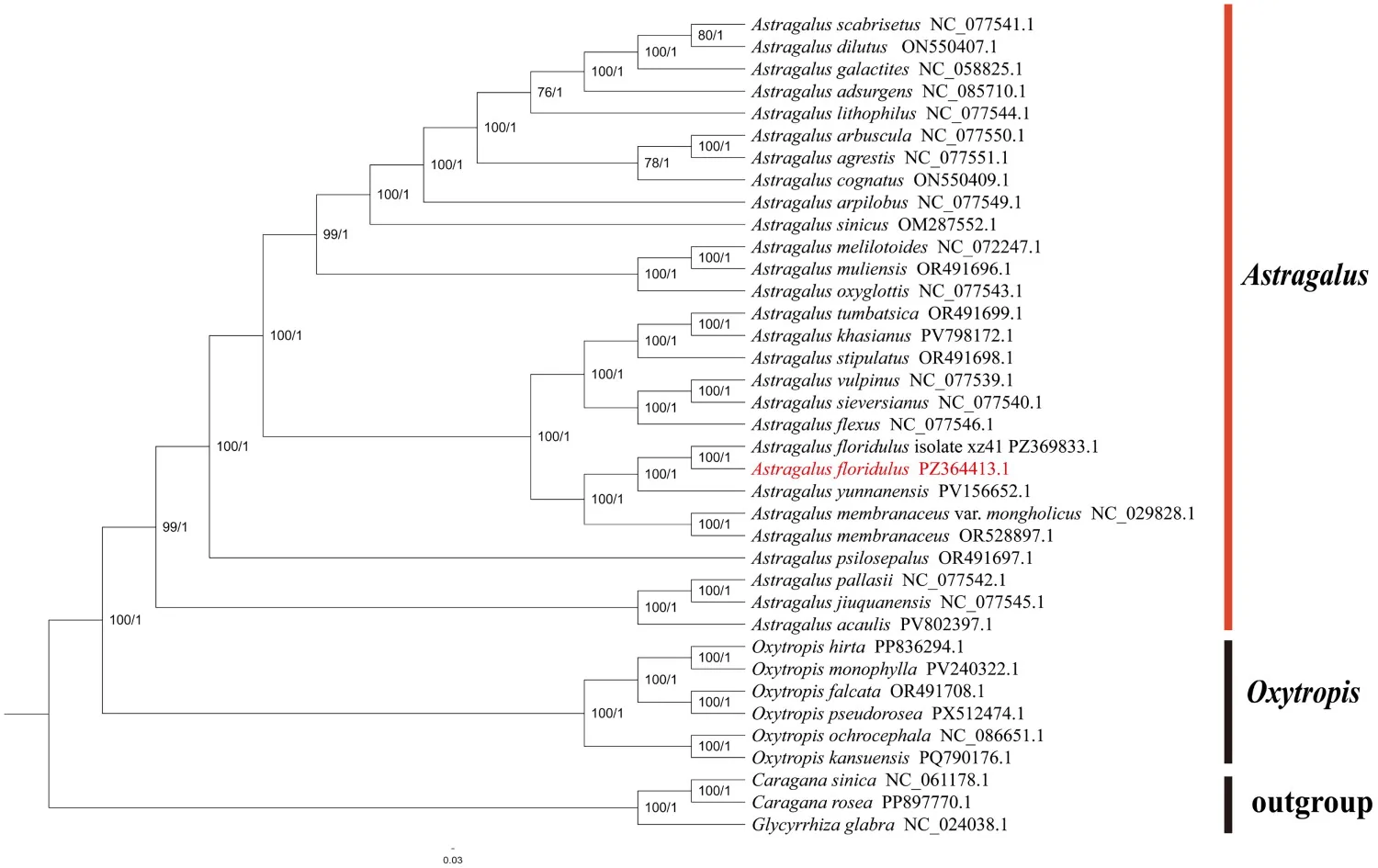
Phylogenetic tree inferred by ML and BI methods based on 37 complete chloroplast genome accessions representing 36 Fabaceae species. Numbers at nodes represent support values: ML bootstrap (BS) on the left and bayesian posterior probability (PP) on the right. The sequences used for constructing the phylogenetic tree are as follows: *Astragalus scabrisetus* NC_077541.1, *A. dilutus* ON550407.1, *A. galactites* NC_058825.1, *A. adsurgens* NC_085710.1 (Tian et al. 2021), *A. lithophilus* NC_077544.1, *A. arbuscula* NC_077550.1, *A. agrestis* NC_077551.1, *A. cognatus* ON550409.1, *A. arpilobus* NC_077549.1, *A. sinicus* OM287552.1 (Ke et al. 2022), *A. melilotoides* NC_072247.1 (Tian et al. 2021), *A. muliensis* OR491696.1, *A. oxyglottis* NC_077543.1, *A. tumbatsica* OR491699.1, *A. khasianus* PV798172.1 (Chen et al. 2025), *A. stipulatus* OR491698.1, *A. vulpinus* NC_077539.1, *A. sieversianus* NC_077540.1, *A. flexus* NC_077546.1, *A. floridulus* isolate xz41 PZ369833.1, *A. floridulus* PZ364413.1, *A. yunnanensis* PV156652.1 (Ma et al. 2025), *A. membranaceus* var. *mongholicus* NC_029828.1 (Lei et al. 2016), *A. membranaceus* OR528897.1, *A. psilosepalus* OR491697.1, *A. pallasii* NC_077542.1, *A. jiuquanensis* NC_077545.1, *A. acaulis* PV802397.1, *Oxytropis hirta* PP836294.1, *O. monophylla* PV240322.1, *O. falcata* OR491708.1, *O. pseudorosea* PX512474.1, *O. ochrocephala* NC_086651.1, *O. kansuensis* PQ790176.1 (Lei et al. 2025), *Caragana sinica* NC_061178.1, *C. rosea* PP897770.1, *Glycyrrhiza glabra* NC_024038.1. The chloroplast genome of *Astragalus floridulus* in this study is labeled in red. Scale bar indicates 0.03 nucleotide substitutions per site.

## 4. Discussion and conclusion

As one of the three major plant genomes, the chloroplast genome is characterized by its conserved structure, moderate evolutionary rate, maternal inheritance, and low recombination rate (Li et al. 2024), and has been widely employed in species identification, phylogenetic analysis, and elucidation of species origins (Qian et al. 2025). Cis-spliced genes have coding exons on the same precursor mRNA, joined via intramolecular splicing (Falcon de Longevialle et al. 2010), whereas trans-spliced genes have exons on separate pre-mRNA molecules, ligated via intermolecular splicing (Hildebrand et al. 1988; Sutton and Boothroyd 1986).

The chloroplast genome of *Astragalus floridulus* exhibits the characteristic IRLC architecture, with the loss of one copy of the canonical inverted repeat. This is consistent with the chloroplast genome features previously reported for most *Astragalus* species (Chen et al. 2025; Ke et al. 2022; Lei et al. 2016; Ma et al. 2025; Tian et al. 2021). The loss of one IR region is a known phylogenetic feature of the IRLC and may provide useful information for future studies of chloroplast genome structural evolution and phylogenetic relationships in *Astragalus*.

Our phylogenetic analysis confirmed *Astragalus* and *Oxytropis* as distinct monophyletic sister clades, in agreement with previous molecular systematic studies (Lei et al. 2025; Qian et al. 2025). The *A. floridulus* isolate xz41 was collected from Batang County, Sichuan Province, whereas our sample originated from Kangding City within the same prefecture. Despite different geographic origins, chloroplast phylogeny supported their assignment to the same species (BS = 100%, PP = 1.0, Figure 3). The two *A. floridulus* accessions and *A. yunnanensis* formed a well-supported monophyletic subclade (BS = 100%, PP = 1.0, Figure 3), suggesting a close relationship. Morphologically, the two species share axillary racemes with secund flowers, a campanulate calyx, leaves glabrous above and pubescent below, and pubescent stipitate ovaries and pods, but are distinguished by stem length, flower number per inflorescence, and pod morphology and size (Xu and Podlech 2010).

Chloroplast genome data remain scarce for many *Astragalus* species, and intrageneric phylogenetic relationships have not been fully resolved. This study sequenced, assembled and annotated the complete chloroplast genome of *Astragalus floridulus* and clarified its phylogenetic position within the genus. These findings fill a gap in *A. floridulus* genomic research, enrich the *Astragalus* chloroplast genome database, and provide foundational data for species identification, germplasm conservation and medicinal resource utilization.

## Supplementary Material

supplementary material clean copy.docx

## Data Availability

The genome sequence data that support the findings of this study are openly available in GenBank (NCBI, https://www.ncbi.nlm.nih.gov/) under the accession number PZ364413.1. The associated BioProject, SRA, and BioSample numbers are PRJNA1457211, SRR38355800, and SAMN57467603, respectively.
