# Supplementary material for: The complete chloroplast genome and phylogenetic analysis of Astragalus floridulus Podlech 1993 (Fabaceae)
Source: Mitochondrial DNA B Resour. 2026 Nov 2;11(12):1378–83. doi: 10.1080/23802359.2026.2753255 (PMC13729629; doi:10.1080/23802359.2026.2753255)
Supplement: Supplementary file 1 [file TMDN_A_2753255_SM6592.docx]

**Figure S1.** The map of sequencing depth and coverage, representing the sequencing depth across the chloroplast genome. The x-axis shows the genomic position (in base pairs, bp), and the y-axis shows the sequencing depth (×). The blue line indicates the sequencing depth at each genomic position.

**Figure S2.** The map of the cis-splicing genes includes ten genes (*ycf3*, *rpoC1*, *atpF*, *clpP*, *petB*, *petD*, *rpl16*, *rpl2*, *ndhB* and *ndhA*). Nine of them contain one intron and two exons, whereas *ycf3* contains two introns and three exons.

**Figure S3.** The map of the trans-splicing gene *rps12* in the *Astragalus floridulus* chloroplast genome.

**Table S1.** The chloroplast-encoded genes of *Astragalus floridulus*.

| Category | Gene group | Gene name |
| --- | --- | --- |
| Photosynthesis | Subunits of photosystem I | *psaA, psaB, psaC, psaI, psaJ* |
|  | Subunits of photosystem II | *psbA, psbB, psbC, psbD, psbE, psbF, psbH, psbI, psbJ, psbK, psbL, psbM, psbN, psbT, psbZ* |
|  | Subunits of NADH dehydrogenase | *ndhA*, ndhB*, ndhC, ndhD, ndhE, ndhF, ndhG, ndhH, ndhI, ndhJ, ndhK* |
|  | Subunits of cytochrome b/f complex | *petA, petB*, petD*, petG, petL, petN* |
|  | Subunits of ATP synthase | *atpA, atpB, atpE, atpF*, atpH, atpI* |
|  | Large subunit of rubisco | *rbcL* |
| Self-replication | Proteins of large ribosomal subunit | *rpl14, rpl16*, rpl2*, rpl20, rpl23, rpl32, rpl33, rpl36* |
|  | Proteins of small ribosomal subunit | *rps11, rps12*, rps14, rps15, rps18, rps19, rps2, rps3, rps4, rps7, rps8* |
|  | Subunits of RNA polymerase | *rpoA, rpoB, rpoC1*, rpoC2* |
|  | Ribosomal RNAs | *rrn16S, rrn23S, rrn4.5S, rrn5S* |
|  | Transfer RNAs | *trnA-UGC*, trnC-GCA, trnD-GUC, trnE-UUC, trnF-GAA, trnG-GCC, trnG-UCC*, trnH-GUG, trnI-CAU, trnI-GAU*, trnK-UUU*, trnL-CAA, trnL-UAA*, trnL-UAG, trnM-CAU, trnN-GUU, trnP-UGG, trnQ-UUG, trnR-ACG, trnR-UCU, trnS-GCU, trnS-GGA, trnS-UGA, trnT-GGU, trnT-UGU, trnV-GAC, trnV-UAC*, trnW-CCA, trnY-GUA, trnfM-CAU* |
| Other genes | Maturase | *matK* |
|  | Protease | *clpP** |
|  | Envelope membrane protein | *cemA* |
|  | Acetyl-CoA carboxylase | *accD* |
|  | c-type cytochrome synthesis gene | *ccsA* |
| Genes of unknown function | Conserved hypothetical chloroplast ORF | *ycf1, ycf2, ycf3**, ycf4* |

Note: Asterisks indicate intron-containing genes. Gene*: Gene with one intron; Gene**: Gene with two introns.
